# Direct Identification of Acetaldehyde Formation and Characterization of the Active Site in the [VPO_4_]^.+^/C_2_H_4_ Couple by Gas‐Phase Vibrational Spectroscopy

**DOI:** 10.1002/anie.201911040

**Published:** 2019-11-08

**Authors:** Ya‐Ke Li, Sreekanta Debnath, Maria Schlangen, Wieland Schöllkopf, Knut R. Asmis, Helmut Schwarz

**Affiliations:** ^1^ Institut für Chemie Technische Universität Berlin Straße des 17. Juni 135 10623 Berlin Germany; ^2^ Wilhelm-Ostwald Institut für Physikalische und Theoretische Chemie Universität Leipzig Linnéstr. 2 04103 Leipzig Germany; ^3^ Fritz-Haber-Institut der Max-Plank-Gesellschaft Faradayweg 4–6 14195 Berlin Germany

**Keywords:** active site, cryogenic ion trap, heteronuclear oxide clusters, infrared photodissociation spectroscopy, olefin oxidation

## Abstract

The gas‐phase reaction of the heteronuclear oxide cluster [VPO_4_]^.+^ with C_2_H_4_ is studied under multiple collision conditions at 150 K using cryogenic ion‐trap vibrational spectroscopy combined with electronic structure calculations. The exclusive formation of acetaldehyde is directly identified spectroscopically and discussed in the context of the underlying reaction mechanism. In line with computational predictions it is the terminal P=O and not the V=O unit that provides the oxygen atom in the barrier‐free thermal C_2_H_4_→CH_3_CHO conversion. Interestingly, in the course of the reaction, the emerging CH_3_CHO product undergoes a rather complex intramolecular migration, coordinating eventually to the vanadium center prior to its liberation. Moreover, the spectroscopic structural characterization of neutral C_2_H_4_O deserves special mentioning as in most, if not all, ion/molecule reactions, the neutral product is usually only indirectly identified.

## Graphical abstract

**Caught in the act**: The gas‐phase reaction of the heteronuclear oxide cluster [VPO_4_]^.+^ with C_2_H_4_ was studied by infrared photodissociation spectroscopy, which directly identified the formation of acetaldehyde and revealed the terminal P=O moiety as the active site of [VPO_4_]^.+^ in the oxygen atom transfer (OAT) reaction with ethylene, in line with theoretical predictions.

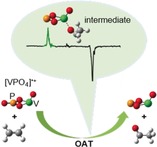

The identification of the active site(s) of single‐site catalysts—the so‐called “aristocratic atoms”^1^—constitutes one of the intellectual cornerstones in contemporary catalysis research.^2^ This holds true in particular for heteronuclear cluster oxides whose judicious “doping” allows for an unprecedented control of their gas‐phase ion chemistry.^3^ In this respect, the redox couple [AlVO_*x*_]^.+^/CO/N_2_O (*x*=3, 4) may serve as a good example.^4^ At room temperature, [AlVO_4_]^.+^ is reduced to [AlVO_3_]^.+^ in the presence of CO, and if N_2_O is added, re‐oxidation occurs; both oxygen atom transfers (OATs) are clean and proceed with reaction efficiencies of 59 and 65 %, respectively. DFT calculations have provided insight into the mechanism of this OAT catalytic cycle and predicted the terminal Al−O and not the expected V−O unit as the active site of the catalyst.^4^ Experimental confirmation of these predictions for both the structure of the [AlVO_4_]^.+^ cluster oxide as well as its active site was only obtained much later by cryogenic ion‐trap vibrational spectroscopy of messenger‐tagged cluster ions.^5^, ^6^

Herein, we describe the spectroscopic characterization of the reaction of the [VPO_4_]^.+^/C_2_H_4_ couple. This heteronuclear oxo cluster has served as a model system of industrially important VPO catalysts^7^ in the gas‐phase oxidation of small hydrocarbons.^8^ At room temperature, [VPO_4_]^.+^ brings about the selective conversion of C_2_H_4_ to form C_2_H_4_O.^8^ In line with DFT calculations, IR photodissociation spectroscopy permitted an unambiguous structural assignment of [VPO_4_]^.+^ (**1**). Other isomers considered were not only predicted to lie >100 kJ mol^−1^ higher in energy than **1**, also their calculated IR spectra did not match the experimentally recorded one.^8^ DFT calculations further predicted that it is only the terminal P=O unit that serves as the active site in the course of the C_2_H_4_→C_2_H_4_O conversion. The alternative OAT process involving the terminal V=O group of **1** was calculated to require too much energy to play a role at ambient temperature.^8^

What has been missing thus far is an unambiguous experimental characterization of relevant reaction intermediates and the products, for example, the structural assignment of the [VPO_3_]^.+^ ion and in particular the neutral OAT C_2_H_4_O (Scheme 1). In fact, to the best of our knowledge, in none of the numerous gas‐phase ion/molecule reactions, the neutral product has ever been spectroscopically characterized. Furthermore, and as suggested by a reviewer, in addition to the previous DFT calculations,^8^ a more detailed discussion of the actual mechanism of the C_2_H_4_→CH_3_CHO conversion is indicated. This will be provided further below.

**Scheme 1:**
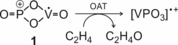
Oxygen atom transfer in the reaction of [VPO_4_]^⋅+^ with C_2_H_4_.

To this end, we revisited the OAT reaction of **1** with C_2_H_4_ and studied it by ion‐trap mass spectrometry in combination with cryogenic ion vibrational spectroscopy (see the Supporting Information for experimental details).^6^, ^9^ Species **1** is produced as previously described,^8^ mass‐selected, and interacts with 0.025 % C_2_H_4_ in He (*p*_He_≈0.022 mbar) under multiple collision conditions in a linear radiofrequency ion trap. The reaction products obtained after 100 ms are shown in Figure 1. Two series of mass peaks are observed. The dominant product channel corresponds to the formation of [VPO_4_,(C_2_H_4_)_*n*_]^.+^ adducts with *n*=0–3. Formation of [VPO_3_]^.+^ and its adducts with C_2_H_4_ is roughly 100 times less efficient. The present results are in qualitative agreement with the previous results obtained under single‐collision conditions, which also found that adduct formation is favored over O‐atom loss; however, adduct formation was less efficient, and obviously no adducts with *n*>1 were observed under single‐collision conditions.^8^ The adduct ion yields are also expected to be higher in the present case as the reaction under scrutiny is predicted to be barrier‐free with respect to the energy of the entrance channel (energy of **1** and C_2_H_4_ in Figure 2), and hence shows a negative temperature dependence.

**Figure 1:**
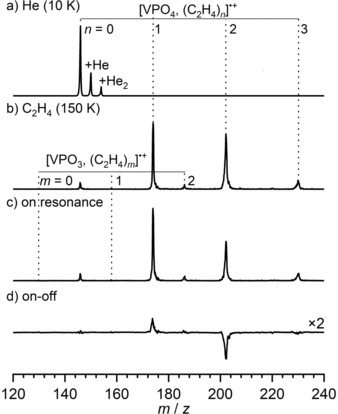
Time‐of‐flight (TOF) mass spectra obtained after storing mass‐selected [VPO_4_]^.+^ ions for 100 ms in the ion trap filled with a) He at 10 K and b) C_2_H_4_/He and held at 150 K. c) Upon resonant excitation ($\bar{\nu }=$ 1655 cm^−1^; see Figure 3), fragmentation of the weaker bound adducts (*n*>1) occurs. d) Difference spectrum, obtained by subtracting the on‐resonance from an off‐resonance ($\bar{\nu }=$ 1685 cm^−1^) mass spectrum, showing the depletion (downward peaks) of the parent ions (*n*>1) and the formation of the corresponding fragment ions (*n*≤1). Note that the ions with *n*≤1 are more strongly bound, making their dissociation less probable.

**Figure 2:**
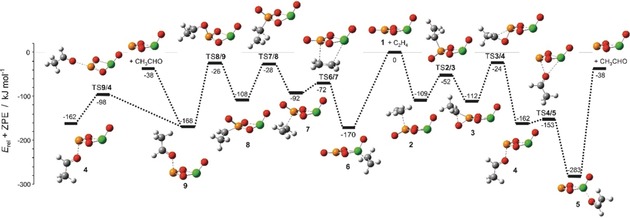
Simplified electronic ground‐state PES for the reactions of [VPO_4_]^.+^ with C_2_H_4_, calculated at the B3LYPD2/def2‐TZVPP level of theory. C gray, H white, V green, O red, P yellow. The relative energies Δ*H*_0K_ are given in kJ mol^−1^ and corrected for unscaled ZPE contributions.

As to the mechanism of OAT in the [VPO_4_]^.+^/C_2_H_4_ couple, the new DFT calculations (Figure 2) provide some unexpected findings:

1. Coordination of the incoming C_2_H_4_ ligand can occur at both the phosphorus (**1**→**2**) and the vanadium sites of the cluster (**1→6**), with the latter path being energetically clearly favored.
2. In the course of the multistep OAT reaction, the global minimum corresponds to a complex in which the newly formed CH_3_CHO ligand (containing the oxygen atom of the P=O unit) is coordinated to the vanadium center (**1**→→→**5**). The isomeric cluster **9**, generated from **6**, is approximately 115 kJ mol^−1^ less stable than **5**. However, as the transition states leading to both **5** and **9** are located below the entrance channel **1**+C_2_H_4_, both routes are energetically accessible. Interestingly, **5** and **9** are connected via transition state **TS9/4** and **TS4/5**.
3. Evaporation of CH_3_CHO to produce [P(O_2_)VO]^.+^ can take place from either **4**, **5**, or **9**.

In order to identify the structure of the reaction product(s), we turn to infrared photodissociation (IRPD) spectroscopy combined with messenger tagging to ensure probing in the linear absorption regime; this simplifies the interpretation of the IRPD spectra significantly.^10^ The IRPD experiments are performed on a cryogenic ion‐trap tandem mass spectrometer^11^ using the widely tunable, intense IR radiation from the Fritz Haber Institute free electron Laser (FHI‐FEL).^12^ As typical messengers, such as He or H_2_, do not bind efficiently to cations at the present ion‐trap temperature of 150 K, we exploit the fact that larger [VPO_4_,(C_2_H_4_)_*n*_]^.+^ adducts are formed, in which the additional C_2_H_4_ moieties are more weakly bound and function as messengers upon photoabsorption. This is demonstrated in the mass spectra shown in the bottom panels of Figure 1. The spectrum in Figure 1 c is obtained after irradiating all ions extracted from the ion trap on‐resonance (1655 cm^−1^), and the difference spectrum (Figure 1 d) is obtained by subtracting an off‐resonance spectrum (1685 cm^−1^) from this on‐resonance spectrum. Using sufficiently attenuated laser pulse energies, photodissociation is only observed for the *n*>1 adducts, that is, the corresponding peaks are depleted in the on‐resonance spectrum (i.e., downward peaks in Figure 1 d), while the *n*≤1 peaks increase in intensity (i.e., upward peaks in Figure 1 d), suggesting that [VPO_4_,C_2_H_4_]^.+^⋅(C_2_H_4_)_*n*−1_ type complexes are present, containing a single, chemically transformed ethylene species, while the others remain physisorbed.

The IRPD spectrum of [VPO_4_,C_2_H_4_]^.+^⋅(C_2_H_4_) is shown in Figure 3 b and compared to the previously obtained spectrum of [VPO_4_]^.+^⋅He_2_ (Figure 3 a).^8^ The two spectra are distinctly different, showing that a reaction must have occurred. Note that the terminal P=O stretching band of [VPO_4_]^.+^ at 1445 cm^−1^ is not present in the spectrum of the C_2_H_4_ adduct, but has been replaced by a similarly intense band at higher energies (1653 cm^−1^), which lies in the carbonyl stretching region.

**Figure 3:**
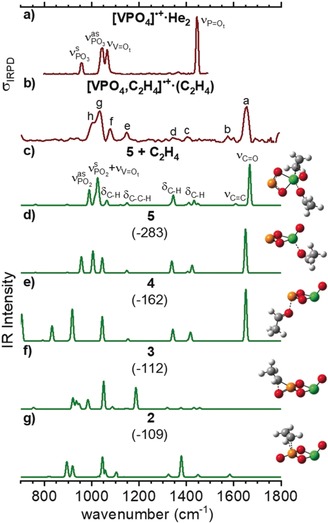
Experimental IRPD spectra (dark red) of a) [VPO_4_]^.+^⋅He_2_ at 15 K,^8^ b) [VPO_4_,C_2_H_4_]^.+^⋅(C_2_H_4_) at 150 K, and the harmonic B3LYPD2/def2‐tzvpp IR spectra (green, Gaussian line function convolution FWHM=10 cm^−1^) of c) **5**+C_2_H_4_, d) **5**, e) **4**, f) **3**, and g) **2**. C gray, H white, V green, O red, P yellow. The zero‐point vibration‐corrected energies (Δ*H*_0K_, shown in parentheses) with respect to the separated reactants are given in kJ mol^−1^. Harmonic frequencies of the V=O_t_ modes are scaled by 0.9167 and all other modes by 0.9832. See Table 1 for the band positions and assignments. Note that the calculated IR spectra of **4** and **9** (not shown) are practically indistinguishable.

In order to assign the IRPD spectrum of [VPO_4_,C_2_H_4_]^.+^⋅ (C_2_H_4_), we compare it to the B3LYPD2/def2‐tzvpp harmonic spectra of possible structural candidates in Figure 3 (see Figures S1 and S2 in the Supporting Information for additional information). Indeed, the best agreement is found for the global minimum‐energy structure **5**, which is predicted to lie 283 kJ mol^−1^ below the entrance channel and represents the final reaction product containing acetaldehyde bound to [P(O)_2_VO]^.+^. The exclusive formation of **5** rather than **4** or **9** (Figure 3 b, d, and e, respectively) as the long‐lived [VPO_3_]^.+^/CH_3_CHO intermediate in the ion trap is quite remarkable; after all, the OAT potential energy surface is rather complex and involves quite a number of isomeric intermediates and transition states. Nevertheless, most likely on thermochemical grounds, the CH_3_CHO product undergoes an intracomplex migration from the P‐ to the V‐center to form **5** (Figure 2). Note that the agreement of the IRPD spectrum with the predicted harmonic spectrum is improved after the second, weakly bound C_2_H_4_ molecule is considered in the calculations (see Table 1 for band assignments), highlighting its small but distinct perturbation. However, if this is considered, the complete IRPD spectrum is reproduced, demonstrating that the formation of other long‐lived intermediates and products with this *m*/*z* ratio is insignificant.

**Table 1:** Experimental band positions (in cm^−1^, see Figure 3 b), scaled harmonic vibrational wavenumbers (in cm^−1^, see Figure 3 c), IR intensities (in parentheses, in km mol^−1^) of **5**+C_2_H_4_, and band assignments.

Band	Exp.	B3LYPD2/def2‐tzvpp	Assignment^[c]^
a	1653	1668^[b]^(360)	ν(C=O)
b	1575	1608^[b]^(13)	ν(C=C)
c	1404	1433^[b]^(43)	δ(C−H) in CH_3_CH=O moiety
d	1347	1345^[b]^(89)	δ(C−H) in CH_3_CH=O moiety
e	1147	1148^[b]^(21)	δ(C−C‐H) in CH_3_CH=O moiety
f	1078	1063^[b]^(41)	δ(C−H) in CH_2_=CH_2_
g	1033	1025^[a,b]^(242)	ν^s^(PO_2_)+ν(*V*=O_t_)
h	999	989^[b]^(136)	ν^as^(PO_2_)

[a] Scaling factor: 0.9167 (VO stretches). [b] Scaling factor: 0.9832 (all other modes). [c] Stretching (ν), bending (δ), symmetric (s), antisymmetric (as).

Using C_2_H_4_ as a messenger tag raises the interesting question as to what the influence of the second C_2_H_4_ molecules is on the reaction pathway. To address this, we also performed experiments under single collision conditions (with respect to ion–ethylene collisions), but did not observe any substantial photodissociation of untagged [VPO_4_,C_2_H_4_]^.+^. Note that the predicted dissociation energy of **5** is 283 kJ mol^−1^, which would amount to the absorption of roughly 20 photons at 1000 cm^−1^, which is unlikely under the present experimental conditions. This shows that for bare [VPO_4_]^.+^, chemisorption of C_2_H_4_ is highly favored over physisorption, and therefore the influence of the second C_2_H_4_ molecule is probably negligible under the present conditions.

Additional and independent spectroscopic support that the P‐center represents the active site for the OAT from [VPO_4_]^.+^ to C_2_H_4_ is found in the IRPD spectrum of another product ion, namely that of (C_2_H_4_)_*n*_‐tagged [VPO_3_]^.+^. Although the yield of [VPO_3_]^.+^⋅(C_2_H_4_)_1–2_ is small compared to that of [VPO_4_,C_2_H_4_]^.+^⋅(C_2_H_4_) (see Figure 1), we were able to record an IRPD spectrum of [VPO_3_]^.+^⋅(C_2_H_4_)_2_. The good agreement between the experimental and computational results (Figure S3) confirms that the P−O_2_−V=O, and not the O=P−O_2_−V structure is generated upon desorption of CH_3_CHO from [VPO_4_,(C_2_H_4_)_2_]^.+^.

In summary, the present study has experimentally confirmed the previously predicted mechanism, which postulated that the P‐atom represents the active site of the heteronuclear cluster [VPO_4_]^.+^ for the reaction of [VPO_4_]^.+^ with C_2_H_4_. Moreover, while in most ion/molecule reactions studied, characterization of the neutral product is based on circumstantial evidence,^13^ in the present case the OAT product was spectroscopically identified as CH_3_CHO.

## Conflict of interest

The authors declare no conflict of interest.

## Supporting information

As a service to our authors and readers, this journal provides supporting information supplied by the authors. Such materials are peer reviewed and may be re‐organized for online delivery, but are not copy‐edited or typeset. Technical support issues arising from supporting information (other than missing files) should be addressed to the authors.

Supplementary
