# Supplementary material for: Direct Identification of Acetaldehyde Formation and Characterization of the Active Site in the [VPO4].+/C2H4 Couple by Gas‐Phase Vibrational Spectroscopy
Source: Angew Chem Int Ed Engl. 2019 Nov 8;58(52):18868–72. doi: 10.1002/anie.201911040 (PMC6973009; doi:10.1002/anie.201911040)
Supplement: Supplementary file 1 — Supplementary [file ANIE-58-18868-s001.pdf]

Angewandte Chemie
Eine Zeitschrift der Gesellschaft Deutscher Chemiker

Supporting Information

Direct Identification of Acetaldehyde Formation and Characterization of the Active Site in the $[VPO_4]^{\cdot+}/C_2H_4$ Couple by Gas-Phase Vibrational Spectroscopy

Ya-Ke Li, Sreekanta Debnath, Maria Schlangen, Wieland Schöllkopf, Knut R. Asmis, and Helmut Schwarz**

anie_201911040_sm_miscellaneous_information.pdf

Supporting Information

1) **Experimental methods section**
2) **Computational section**
3) **Details regarding the structures in Figure 3**
4) **IRPD Spectroscopy of $[VPO_4, (C_2H_4)_2]^{\bullet+}$**
5) **RPD Spectroscopy of $[VPO_3, (C_2H_4)_2]^{\bullet+}$**
6) **References**

1) Experimental methods section

The infrared photodissociation (IRPD) experiments are performed on a cryogenic ion trap tandem mass spectrometer[1] using the widely tunable, intense IR radiation from the Fritz-Haber-Institute Free−Electron Laser (FHI FEL).[2] $[VPO_4]^{\bullet+}$ ions are generated as previously described.[3] Briefly, $[VPO_4]^{\bullet+}$ ions are produced by collision induced dissociation after the corresponding precursor is transferred to the gas phase using a commercial Z−spray electrospray ionization (ESI) source and a millimolar solution of $VOCl_3$ and $PO(OEt)_3$ (both purchased from Sigma−Aldrich) in methanol, which were introduced through a stainless steel capillary to the ESI source by a syringe pump (~10 µL min^{-1}). Nitrogen was used as nebulizing and drying gas at a source temperature of 80 °C. The best ion yield was achieved by adjusting the cone voltage (UC) to 170 V. The beam of cations pass through a 4 mm diameter skimmer and are collimated in a radio frequency (RF) decapole ion guide. The desired cations are mass−selected using a quadrupole mass filter, deflected 90° in an electrostatic quadrupole deflector, and focused into a cryogenic RF ring−electrode ion trap. The trap is continuously filled with a reactant gas/ buffer gas mix of 0.025 % C_2H_4 in He at an ion-trap temperature of 150 K. Many collisions of the trapped ions with the mixture gas provide gentle cooling of the internal degrees of freedom close to the ambient temperature. Under these conditions, only small amounts of $[VPO_3]^{\bullet+}$ and C_2H_4-tagged $[VPO_3]^{\bullet+}$ ions $[VPO_3]^{\bullet+}\cdot(C_2H_4)_{1\text{-}2}$ (see Fig. 1b and Fig. 3b) are formed.

For the IRPD experiments of the $[VPO_4, C_2H_4]^{\bullet+}$ complex we used a second C_2H_4 molecule as a messenger tag (Fig. 3 and Fig. S2). All ions are extracted from the ion trap at 5 Hz and focused both temporally and spatially into the center of the extraction region of an orthogonally mounted reflection time−of−flight (TOF) tandem photofragmentation mass−spectrometer. Here, the ions are irradiated with a counter−propagating IR laser pulse produced by the FHI FEL (700−1800 cm^{-1}, bandwidth: ~0.5 % fwhm, pulse energy: 4−32 mJ). All parent and photofragment ions are then accelerated toward an MCP detector and monitored simultaneously. IRPD scans are recorded by averaging about 100 TOF mass spectra per wavelength step (3 cm^{-1}) and scanning the wavelength. Typically, at least three scans are summed to obtain the final IRPD spectrum. The photodissociation cross section σ_{IRPD} is determined as described previously.[4]

2) Computational section

All calculations were performed using the Gaussian09 package.[5] Geometries were optimized at the unrestricted UB3LYPD2 level of theory[6] with the triple-ζ plus polarization basis sets def2-TZVP.[7] Vibrational frequency analyses have been carried out at the same level of theory to characterize the nature of stationary points as minima or transition structures, to derive the zero-point energy (ZPE)

corrections, and to assign the IR relevant structures. All relative energies presented in this work are corrected for ZPE and given in kJ mol^{-1}. Intrinsic reaction coordinate (IRC)[8] calculations or manual displacements along the reaction trajectory of the imaginary frequency were performed to link all transition states with the respective intermediates. All given ionic reactants, intermediates, and transition states have been located on the ground-state doublet potential energy surface.

It is known that B3LYP vibrational frequencies are systematically too large;[9] however, the agreement with observed frequencies can be improved by appropriate scaling. Scaling then accounts for both anharmonicities and systematic errors of the calculated harmonic force constants (calculated harmonic wavenumbers are compared to observed fundamentals including anharmonicities). We use scaling parameters that were determined for small vanadium oxide cluster cations.[10] The vanadyl (V = O) modes are scaled by 0.9167 and all other modes by 0.9832. The simulated linear absorption spectra are derived from scaled harmonic frequencies and intensities.

3) Details regarding the structures in Figure 3

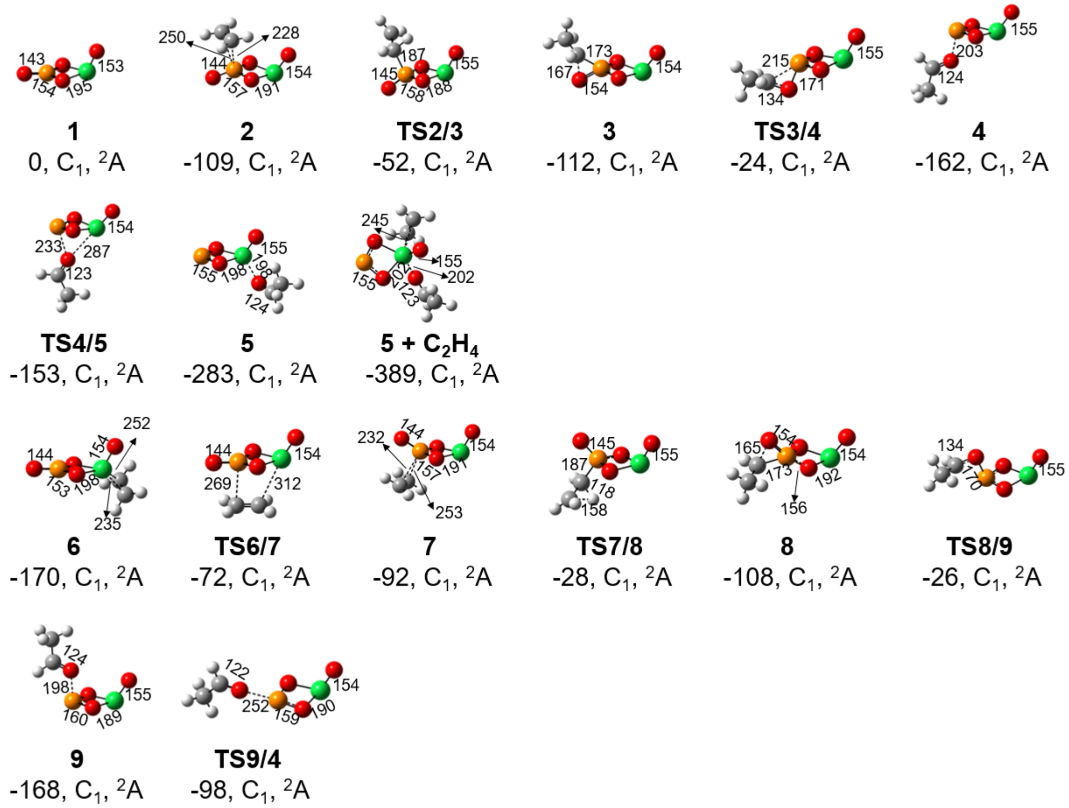

Figure S1. ZPE-corrected energy (ΔH_{0K}) with respect to the separated reactants (kJ mol^{-1}), symmetry, electronic state and bond lengths (pm) of the B3LYPD2/def2−tzvpp structures shown in Figures 2 and 3 (color code: yellow, P; green, V; red, O; gray, C; white, H).

4) IRPD Spectroscopy of $[VPO_4, (C_2H_4)_2]^{•+}$

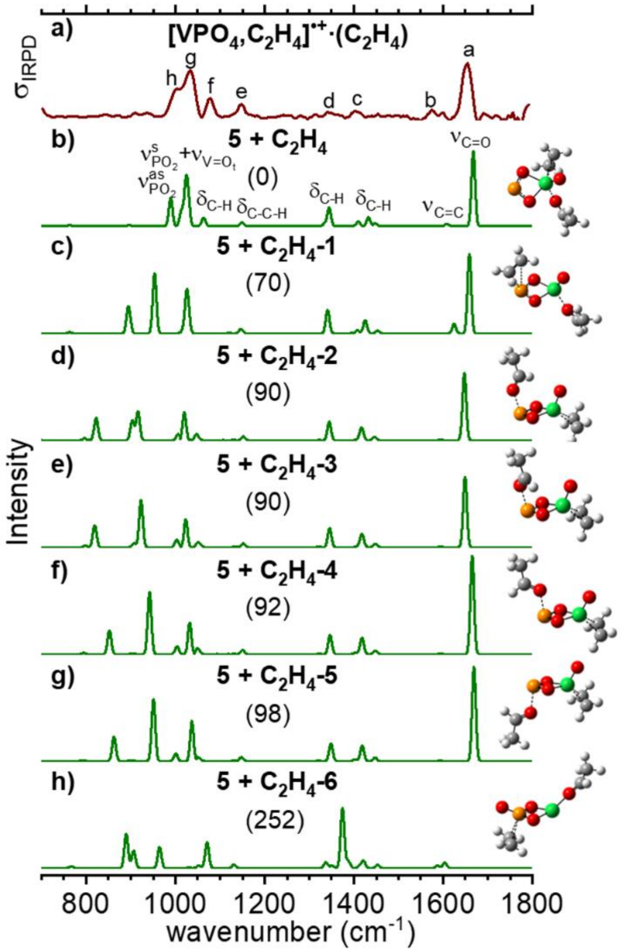

Figure S2 Experimental IRPD spectra (dark red) of $[VPO_4,C_2H_4]^{•+}·(C_2H_4)$ at 150 K compared with calculated harmonic B3LYPD2/def2−tzvpp spectra (green, Gaussian line function convolution, FWHM=10 cm^{-1}) of various isomers. Numbers in parenthesis indicates relative ZPE−corrected energies (ΔH_{0K}, in kJ mol^{-1}) of the isomers (yellow, P; green, V; red, O; gray, C; white, H). Harmonic frequencies of the $V=O_t$ modes are scaled by 0.9167 and all other modes by 0.9832.

The band at 1653 cm^{-1} is assigned to the C=O stretching mode. The weak band b (1575 cm^{-1}) is assigned to the C=C stretching mode, which confirms that the integrity of the second ethylene, which acts as a tag, remains intact. The other two intense bands g (1033 cm^{-1}) and h (999 cm^{-1}) are assigned to the combination of symmetric PO_2 stretching with terminal $V=O_t$ stretching mode and antisymmetric PO_2 stretching mode, respectively. The terminal $V=O_t$ stretching mode at 1033 cm^{-1} is line with the IRPD data reported previously for $[CeVO_4]^{•+}$,[11] $[V_2O_4]^{•+}$,[10] $VPO_4]^{•+}$,[3] and $[AlVO_4]^{•+}$.[12] The weak bands c (1404 cm^{-1}), d (1347 cm^{-1}), e (1147 cm^{-1}) are assigned to two C–H bending modes and the C–C–H bending mode in $CH_3CH=O$ moiety, respectively, and the weak band f (1078 cm^{-1}) is assigned to the C–H bending mode in $CH_2=CH_2$.

5) IRPD Spectroscopy of $[VPO_3, (C_2H_4)_2]^{\bullet+}$

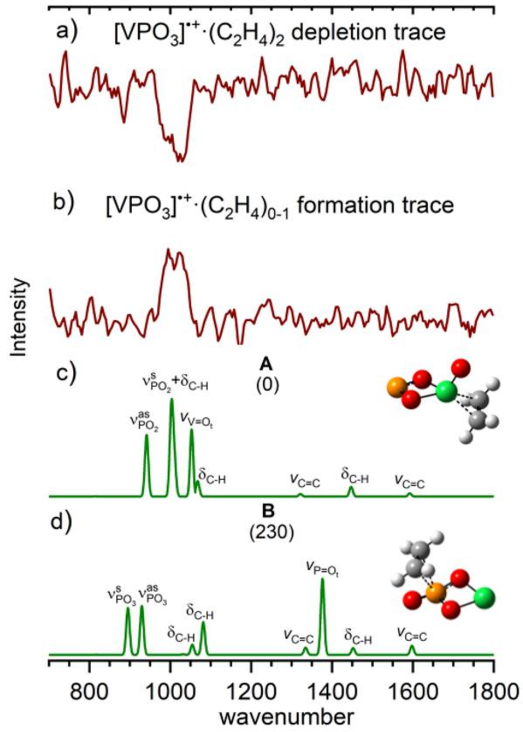

Figure S3. Experimental (dark red) (a) IRPD depletion trace of $[[VPO_3]^{\bullet+}\cdot(C_2H_4)_2$, (b) IRPD formation trace of $[VPO_3]^{\bullet+}\cdot(C_2H_4)_{0,1}$ at 150 K, and the harmonic B3LYPD2/def2−tzvpp IR spectra (green, Gaussian line function convolution FWHM=10 cm^{-1}) of structure c) **A** ($PO_2VO{-}C_2H_4$) and d) **B** ($C_2H_4{-}OPO_2V$) (color code: yellow, P; green, V; red, O; gray, C; white, H). The relative, ZPE-corrected energies (ΔH_{0K}, shown in parentheses) are given in kJ mol^{-1}. Harmonic frequencies of the V=O$_t$ modes are scaled by 0.9167 and all other modes by 0.9832.

The reaction of $[VPO_4]^{\bullet+}$ with C_2H_4 also yields small amounts of C_2H_4-tagged $[VPO_3]^{\bullet+}$ ions, which are formed by CH_3CHO desorption from $[VPO_4,C_2H_4]^{\bullet+}\cdot(C_2H_4)_{n,}$. We also measured IRPD spectra of these ions. The comparison in Fig. S3 shows that C_2H_4-tagged $[VPO_3]^{\bullet+}$ ions contain the P–O$_2$–V=O, and not the O=P–O$_2$–V, structure, yielding additional support that the P-center represents the active site for the OAT reaction of $[VPO_4]^{\bullet+}$ with C_2H_4.

6) References

[1] a) D. J. Goebbert, T. Wende, R. Bergmann, G. Meijer, K. R. Asmis, *J. Phys. Chem. A* **2009**, *113*, 5874-5880; b) D. J. Goebbert, G. Meijer, K. R. Asmis, *AIP Conf. Proc.* **2009**, *1104*, 22-29.

[2] W. Schöllkopf, S. Gewinner, H. Junkes, A. Paarmann, G. von Helden, H. Bluem, A. M. M. Todd, in *Advances in X-Ray Free-Electron Lasers Instrumentation Iii, Vol. 9512* (Ed.: S. G. Biedron), Spie-Int Soc Optical Engineering, Bellingham, **2015**.

[3] N. Dietl, T. Wende, K. Chen, L. Jiang, M. Schlangen, X. Zhang, K. R. Asmis, H. Schwarz, *J. Am. Chem. Soc.* **2013**, *135*, 3711-3721.

[4] a) N. Heine, K. R. Asmis, *Int. Rev. Phys. Chem.* **2016**, *35*, 507-507; b) N. Heine, K. R. Asmis, *Int. Rev. Phys. Chem.* **2015**, *34*, 1-34.

[5] M. J. Frisch, G. W. Trucks, H. B. Schlegel, G. E. Scuseria, M. A. Robb, J. R. Cheeseman, G. Scalmani, V. Barone, B. Mennucci, G. A. Petersson, H. Nakatsuji, M. Caricato, X. Li, H. P. Hratchian, A. F. Izmaylov, J. Bloino, G. Zheng, J. L. Sonnenberg, M. Hada, M. Ehara, K. Toyota, R. Fukuda, J. Hasegawa, M. Ishida, T. Nakajima, Y. Honda, O. Kitao, H. Nakai, T. Vreven, J. A. M. Jr., J. E. Peralta, F. Ogliaro, M. Bearpark, J. J. Heyd, E. Brothers, K. N. Kudin, V. N. Staroverov, R. Kobayashi, J. Normand, K. Raghavachari, A. Rendell, J. C. Burant, S. S. Iyengar, J. Tomasi, M. Cossi, N. Rega, J. M. Millam, M. Klene, J. E. Knox, J. B. Cross, V. Bakken, C. Adamo, J. Jaramillo, R. Gomperts, R. E. Stratmann, O. Yazyev, A. J. Austin, R. Cammi, C. Pomelli, J. W. Ochterski, R. L. Martin, K. Morokuma, V. G. Zakrzewski, G. A. Voth, P. Salvador, J. J. Dannenberg, S. Dapprich, A. D. Daniels, Ö. Farkas, J. B. Foresman, J. V. Ortiz, J. Cioslowski, D. J. Fox, Gaussian 09, Revision D.01, Gaussian, Inc.: Wallingford CT, **2009**.

[6] a) C. T. Lee, W. T. Yang, R. G. Parr, *Physical Review B* **1988**, *37*, 785-789; b) A. D. Becke, *J. Chem. Phys.* **1993**, *98*, 5648-5652; c) S. Grimme, *J. Comput. Chem.* **2006**, *27*, 1787-1799.

[7] A. Schafer, C. Huber, R. Ahlrichs, *J. Chem. Phys.* **1994**, *100*, 5829-5835.

[8] a) K. Fukui, *Acc. Chem. Res.* **1981**, *14*, 363-368; b) D. G. Truhlar, M. S. Gordon, *Science* **1990**, *249*, 491-498; c) C. Gonzalez, H. B. Schlegel, *J. Phys. Chem.* **1990**, *94*, 5523-5527.

[9] a) A. P. Scott, L. Radom, *J. Phys. Chem.* **1996**, *100*, 16502-16513; b) M. D. Halls, J. Velkovski, H. B. Schlegel, *Theor. Chem. Acc.* **2001**, *105*, 413-421.

[10] L. Jiang, T. Wende, P. Claes, S. Bhattacharyya, M. Sierka, G. Meijer, P. Lievens, J. Sauer, K. R. Asmis, *J. Phys. Chem. A* **2011**, *115*, 11187-11192.

[11] K. R. Asmis, G. Meijer, M. Brümmer, C. Kaposta, G. Santambrogio, L. Woste, J. Sauer, *J. Chem. Phys.* **2004**, *120*, 6461-6470.

[12] S. Debnath, H. Knorke, W. Schöllkopf, S. D. Zhou, K. R. Asmis, H. Schwarz, *Angew. Chem. Int. Ed.* **2018**, *57*, 7448-7452.
